# Pharmacokinetic Differences of Wuji Pill Components in Normal and Chronic Visceral Hypersensitivity Irritable Bowel Syndrome Rats Attributable to Changes in Tight Junction and Transporters

**DOI:** 10.3389/fphar.2022.948678

**Published:** 2022-07-08

**Authors:** Zipeng Gong, Qing Yang, Yajie Wang, Xiaogang Weng, Yujie Li, Yu Dong, Xiaoxin Zhu, Ying Chen

**Affiliations:** ^1^ State Key Laboratory of Functions and Applications of Medicinal Plants, Guizhou Provincial Key Laboratory of Pharmaceutics, Guizhou Medical University, Guiyang, China; ^2^ Institute of Chinese Materia Medica, China Academy of Chinese Medical Sciences, Beijing, China; ^3^ Guang’An Men Hospital, China Academy of Chinese Medical Sciences, Beijing, China

**Keywords:** Wuji pill, irritable bowel syndrome, pharmacokinetic differences, tight junction, transporters

## Abstract

The Wuji pill, also called Wuji Wan (WJW), is an effective traditional medicine for the clinical treatment of irritable bowel syndrome (IBS). It is principally composed of *Rhizoma Coptidis*, *Fructus Evodiae Rutaecarpae*, and *Radix Paeoniae Alba*. There have been no reports on the pharmacokinetics of WJW on IBS. Because it is more meaningful to study pharmacokinetics in relation to specific pathological conditions, our study investigated the pharmacokinetic differences of five representative components (berberine, palmatine, evodiamine, rutaecarpine, and paeoniflorin) in normal rats and chronic visceral hypersensitivity IBS (CVH-IBS) model rats after single dose and multiple doses of WJW using ultra-performance liquid chromatography tandem mass spectrometry (UPLC-MS/MS). Transmission electron microscopy, immunohistochemistry, and immunofluorescence were used to explore mechanisms behind the pharmacokinetic differences in terms of tight junction proteins (Occludin and ZO-1), myosin light chain kinase (MLCK), and transporters including P-glycoprotein (P-gp), multidrug resistance associated protein 1 (MRP1), and multidrug resistance associated protein 2 (MRP2) in rat colons. After a single dose, for all components except rutaecarpine, significant differences were observed between normal and model groups. Compared with normal group, T_1/2_ and AUC_0-t_ of berberine and palmatine in model group increased significantly (562.5 ± 237.2 vs. 1,384.9 ± 712.4 min, 733.8 ± 67.4 vs. 1,532.4 ± 612.7 min; 5,443.0 ± 1,405.8 vs. 9,930.8 ± 2,304.5 min·ng/ml, 2,365.5 ± 410.6 vs. 3,527.0 ± 717.8 min·ng/ml), while Cl/F decreased (840.7 ± 250.8 vs. 397.3 ± 142.7 L/h/kg, 427.7 ± 89.4 vs. 288.9 ± 114.4 L/h/kg). C_max_ and AUC_0-t_ of evodiamine in model group increased significantly (1.4 ± 0.6 vs. 2.4 ± 0.7 ng/ml; 573 ± 45.3 vs. 733.9 ± 160.2 min·ng/ml), while T_1/2_, T_max_, Cl/F, and Vd/F had no significant difference. T_max_ and AUC_0-t_ of paeoniflorin in model group increased significantly (21.0 ± 8.2 vs. 80.0 ± 45.8 min; 15,428.9 ± 5,063.6 vs. 33,140.6 ± 5,613.9 min·ng/ml), while Cl/F decreased (110.5 ± 48.1 vs. 43.3 ± 9.5 L/h/kg). However, after multiple doses, all five components showed significant differences between normal and model groups. Moreover, these differences were related to tight junction damage and the differential expression of transporters in the colon, suggesting that dose adjustment might be required during administration of WJW in the clinical treatment of IBS.

## 1 Introduction

Irritable bowel syndrome (IBS) is a type of intestinal dysfunction with no apparent abnormality in gastrointestinal structure. It is characterized by repeated abdominal pain and changes in intestinal function. Its clinical symptoms include abdominal pain, diarrhea, constipation, or alternating occurrence of constipation, and diarrhea. Previous studies have indicated gastrointestinal motility and sensory disorders resulting from a variety of central and peripheral mechanisms—as the cause of IBS (Kassam et al., 2013; Öhman et al., 2015), but the specific pathogenesis of the disease is still unclear. The involvement of more than one factor is possible. Clinically, colonic dyskinesia and chronic visceral hypersensitivity are the chief manifestations of IBS. As the pathogenesis of IBS is still not clear, treatment is largely targeted at symptoms with limited success. Traditional Chinese medicine (TCM), however, has gained global attention because of its accurate clinical effect.

The Wuji pill, also known as Wuji Wan (WJW), is a classic medicine for the treatment of IBS. It is included in the 1977 edition of Chinese Pharmacopoeia. This medicine has a simple composition (*Rhizoma Coptidis*, *Fructus Evodiae Rutaecarpae*, and *Radix Paeoniae Alba*), and is mainly used to relieve symptoms such as abdominal pain, diarrhea, vomiting and gastric reflux, burning epigastric pain, mouth bitterness, and gastric discomfort. Earlier medical books differ regarding the prescribed ratio of the three herbs in WJW. The Chinese Pharmacopoeia (2020 edition, Volume I) prescribes the following: *Rhizoma Coptidis* 300 g, *Fructus Evodiae Rutaecarpae* 50 g, and *Radix Paeoniae Alba* 300 g (6:1:6) (Chinese Pharmacopoeia Commission, 2020). Recent pharmacological studies have shown that WJW inhibits colon contraction in guinea pigs (Wang et al., 2007), inhibits CYP1A2 enzyme activity (Weng et al., 2010), significantly reduces nitric oxide content in mice infected by *Helicobacter pylori* (Wu et al., 2006), and has anti-colitic (Cho, et al., 1998; Küpeli,et al., 2002; Kuo,et al., 2004; Lee,et al., 2007) and antithrombotic effects (Sheu et al., 2000; Yu, et al., 2009; Jia and Hu, 2010; Yuan,et al., 2011). It is commonly used in the clinical treatment of belching, nausea, vomiting, mouth bitterness, abdominal pain, diarrhea (Chen et al., 2008a), and gastric ulcer (Chen,et al., 2008b). The chemical composition of WJW is well-understood. The main constituents of *Rhizoma Coptidis*, are alkaloids, namely berberine, palmatine, jatrorrhizine, and coptisine, of which berberine is chief active component and the most abundant, in concentrations as much as 10% (Sun,et al., 2006). The *Fructus Evodiae Rutaecarpae* contains predominantly alkaloids, limonoids, and volatile oils, including evodiamine, rutaecarpine, and dehydrorutaecarpine. *Radix Paeoniae Alba* is another drug used in WJW. Its chemical composition includes paeoniflorin, hydroxypaeoniflorin, and albiflorin, of which paeoniflorin is the dominant component (Wang,et al., 2006).

Compared with the many reports on the pharmacology and chemical composition of WJW, some studies have been published on its pharmacokinetics. In the previous study, the absorption, distribution (Zhang, et al., 2014), metabolism and excretion (Wang, et al., 2011) of five representative components including berberine, palmatine, evodiamine, rutaecarpine, and paeoniflorin in WJW were investigated by perfused rat intestine-liver preparation (Wang, et al., 2012), biotransformation of the cytochrome P450 (CYP) enzymes and intestinal flora (Weng, et al., 2010; Men, et al., 2013). Moreover, the pharmacokinetic study of berberine, palmatine, jatrorrhizine, coptisine, evodiamine, rutacarpine, and paeoniflorin in rat by liquid chromatography-tandem mass spectrometry after oral administration of a Chinese medicine WJW were carried out (Yuan, et al., 2012; Chen, et al., 2015). However, the above pharmacokinetic studies on WJW were all conducted in normal animals, and almost none on the pharmacokinetics of this medicine in relation to specific pathological conditions. In recent years, a growing number of studies have shown that the pharmacokinetic characteristics of TCM are influenced by the pathological state of the body. Physiological and pathological changes affect drug-metabolizing enzymes (DMEs), transporters, cell membrane permeability, and microbial flora in the body, thereby altering the ADME of TCM in the body and the pharmacokinetic parameters (Gong et al., 2015). Since TCM is chiefly applied to bodies in the diseased state, it is more meaningful to study its pharmacokinetic parameters in relation to specific pathological conditions rather than in normal body states.

Previous studies on the pharmacokinetics of WJW have all been carried out in normal animals with a single dose of the medicine, and not under specific pathological states. As the pharmacokinetics of a drug is more clinically relevant when studied under a pathological state than under the normal state, it is more meaningful to explore the pharmacokinetics of WJW in a pathological state. Moreover, the number of times a drug is administered also impacts the its pharmacokinetic behavior. Therefore, our study investigated the pharmacokinetic differences of five representative components of WJW (berberine, palmatine, evodiamine, rutaecarpine, and paeoniflorin). We established a chronic visceral hypersensitivity IBS (CVH-IBS) rat model and used ultra-high performance liquid chromatography-tandem mass spectrometry (UPLC/MS-MS) to compare normal rats and model rats after administration of single and multiple WJW doses. We further explored the origin of the pharmacokinetic differences in terms of transporters and tight junctions, with the intention to aid in the adjustment of multiple WJW doses in the ongoing clinical treatment of IBS.

## 2 Materials and Methods

### 2.1 Animal Subjects

A total of 30 neonatal male SD rats (SPF grade, 5 days old) were purchased from Beijing Vital River Laboratory Animal Technology [Certificate No: SCXK (Beijing) 2012-0001]. They were raised with their mothers under natural lighting conditions and were allowed to feed freely. Weaning took place on day 25, after which they were separated into five rats per cage.

### 2.2 Experimental Instruments

The following instruments were used in the study: percutaneous transluminal coronary angioplasty (PTCA) balloon dilatation catheter (specification: 3.0 × 20, Cordis); 8F catheter (Beijing Wandong Kuli’aite Medical Products); Finesse 325 manual microtome, Histocentre 3 tissue embedding machine, and Excelsior ES tissue processor (Thermo Scientific); BX51 microscope (Olympus); Varistan Gemini automated slide stainer (Thermo Scientific); Pressure gauge (Shanghai Medical Instruments); TB-215D electronic analytical balance [Denver Instruments (Beijing)]; rat fixing frame (Beijing Huamei Plexiglass Products); Waters ACQUITY Xevo TQ-XS UPLC/MS System with Masslynx v4.1 (Waters, United States); MSU225S-000-DU semi-microelectronic analytical balance (Sartorius, Germany); VX- III multi-tube vortexer (Beijing Tarjin Tech); NA-5L hybrid nitrogen generator (Beijing ZTE Technology Development); Micropipettes and Centrifuge 5424R low-temperature high-speed centrifuge (Eppendorf, Germany); KQ250E ultrasonic cleaner (Kunshan Ultrasonic Instruments); Milli-Q Advantage A10 water purification system (Millipore, United States).

### 2.3 Reagents and Drugs

#### 2.3.1 Reagents

The following reagents were purchased from Beijing Bioss Biotechnology: *c-fos* rabbit anti-mouse polyclonal antibody, ZO-1 rabbit anti-mouse polyclonal antibody, Occludin rabbit anti-mouse polyclonal antibody, myosin light chain kinase (MLCK) rabbit anti-mouse polyclonal antibody, P-glycoprotein (P-gp) rabbit anti-mouse polyclonal antibody, multidrug resistance associated protein 1 (MRP1) rabbit anti-mouse polyclonal antibody, multidrug resistance associated protein 2 (MRP2) rabbit anti-mouse polyclonal antibody, goat anti-rabbit immunohistochemistry kit, and hematoxylin. Other reagents used were as follows: glacial acetic acid, analytical grade (Beijing Chemical Works, Batch No. 20100603); osmic acid, acetone, embedding media, uranyl acetate stain, and lead citrate stain (supplied by the Experimental Research Center); potassium permanganate, analytical grade (Beijing Chemical Works, Batch No. 820308); toluidine blue, analytical grade (Sinopharm Chemical Reagents, Batch No. WC20050120); anhydrous ethanol, analytical grade (Beijing Chemical Works, Batch No. 20110); xylene, analytical grade (Sinopharm Chemical Reagents, Batch No. 20106); neutral gum (Sinopharm Chemical Reagents, Batch No. 20120320); hematoxylin (Beyotime Biotechnology, Batch No. C0105); ethylenediamine tetraacetic acid (EDTA), analytical grade (Beijing Chemical Works, Batch No. 840529).

#### 2.3.2 Drugs

Berberine hydrochloride (86.7%, Batch No. 110713-201212), palmatine hydrochloride (86.7%, Batch No. 110732-201108), evodiamine (99.9%, Lot 110802-200606), rutaecarpine (99.9%, Lot 110801-201006), paeoniflorin (96.5%, Lot 110736-201136), diphenhydramine hydrochloride (99.9%, Lot 100066-200807: 99.9%), and geniposide (99.9%, Lot 110749-200714) were purchased from the National Institutes for Food and Drug Control, China. Acetonitrile and methanol, chromatographic grade, were purchased from Fisher, United States. Methanoic acid was of chromatographic grade. Water used in the experiment was purified in our laboratory. Other reagents were of analytical grade.

Herbal samples of *Rhizoma Coptidis* were prepared from the dried rhizomes of the plant grown in Sichuan, China, which were identified by Mr. Jinda Hao, a researcher at the Institute of Chinese Materia Medica, China Academy of Chinese Medical Sciences. The berberine content and palmatine content in the samples were 7.93% and 2.29%, respectively. *Rhizoma Coptidis* extract was prepared by the Pharmacy Department of China–Japan Friendship Hospital *via* water extraction and vacuum drying. It was a solid powder with a yield of 19.33%.

Herbal samples of *Fructus Evodiae Rutaecarpae* were prepared from the dried and nearly ripen fruit of the plant grown in Sichuan, China, which were identified by Mr. Jinda Hao, a researcher at the Institute of Chinese Materia Medica, China Academy of Chinese Medical Sciences. The evodiamine content and rutaecarpine content in the samples were 0.49% and 0.37%, respectively. *Fructus Evodiae Rutaecarpae* extract was prepared by the Pharmacy Department of China–Japan Friendship Hospital *via* water extraction and vacuum drying. It was a solid powder with a yield of 17.7%.

Herbal samples of *Radix Paeoniae Alba* were prepared from the dried roots of the plant grown in Anhui, China, which were identified by Mr. Jinda Hao, a researcher at the Institute of Chinese Materia Medica, China Academy of Chinese Medical Sciences. The paeoniflorin content in the samples was 1.69%. *Radix Paeoniae Alba* extract was prepared by the Pharmacy Department of China–Japan Friendship Hospital *via* water extraction and vacuum drying. It was a solid powder with a yield of 9.37%.

Preparation of WJW was as follows: *Rhizoma Coptidis*, *Fructus Evodiae Rutaecarpae*, and *Radix Paeoniae Alba* in a 6:1:6 ratio was made into a 62.96 mg/ml decoction in purified water using ultrasonic dissolution. The concentration of each herbal component in the decoction was as follows: *Rhizoma Coptidis* extract was 38.4 mg/ml. *Fructus Evodiae Rutaecarpae* extract was 5.88 mg/ml, and *Radix Paeoniae Alba* extract was 18.68 mg/ml.

### 2.4 Methods

#### 2.4.1 Grouping

A total of 30 neonatal 5-day-old male SD rats were divided into the normal group and the model group after 3 days of adaptation, with 15 rats in each group. They were raised with their mothers and allowed to feed freely at 24°C room temperature under alternating light/dark cycles of 12 h each. They were weaned on day 25. Following that, they were kept at five rats per cage. All studies were performed in accordance with the proposals of the Committee for Research and Ethical Issues of the International Association for the Study of Pain and were approved by the Animal Ethics Committee at the Institute of Chinese Materia Medica, China Academy of Chinese Medical Sciences (approval number: 20142001).

#### 2.4.2 Establishment of Chronic Visceral Hypersensitivity IBS Model

##### 2.4.2.1 Balloon PTCA Stimulation of Neonatal Rat Colons

Using a method as described previously (AlChaer,et al., 2000), the anus of 8-day-old male neonatal rats was wiped with warm saline. PTCA balloon coated with liquid paraffin and connected to the pressure gauge and inflation syringe *via* a three-way valve was inserted. For the model group, colon stimulation took place by injecting air into the balloon until the pressure slowly increased to 60 mm·Hg. This was performed for 2 weeks, twice a day, for 1 min each time, with a 30 min interval in between. After each stimulation, the PTCA balloon was wiped with alcohol and saline. Rats in the normal group were held gently in hands every day and their perinea massaged for 1 min. The experiment started at 8:00 a.m. every day to eliminate the influence of biological cycle differences. The rats were weighed once a week.

##### 2.4.2.2 Behavioral Study: Colorectal Dilation in Adult Rats

At the end of the eighth week, the adult rats underwent 24 h of fasting and were fixed on a customized plexiglass frame (20 cm × 6 cm × 9 cm). A liquid paraffin-coated catheter was inserted from the anus until the end of the balloon was 2 cm from the anus. The catheter was then taped to the root of the rat tail with medical tape. The other end of the balloon was connected to a syringe filled with warm saline (37°C ± 0.5°C). The rats were allowed to adapt to the environment for 30 min before the catheter was filled with saline. The threshold values of abdominal lift (rat lifting its abdomen 0.2 cm from the platform) and pelvic lift (rat lifting its pelvic structure 1 cm from the platform) were noted. Each rat was measured three times, with each measurement lasting 10 s separated by an interval of 30 min. The results were represented as mean values.

#### 2.4.3 Sampling and Sample Treatment

At the end of the eighth week and 1 h after colon dilation, five rats in each group were immediately sacrificed. A small section of the colon was taken immediately and transferred to a 2.5% glutaraldehyde solution. A piece 1 mm^3^ in size was cut out for inspection of tight junction morphology under transmission electron microscopy. A section of colon tissue was taken at 2 cm from the anus and another at 2 cm from the ileum–colon junction and fixed in 4% paraformaldehyde solution. The fixed colon tissue went through gradient dehydration with ethanol and was cleared with xylene before paraffin embedding. It was sliced into a thickness of 4 μm and allowed to fully unfold in water at 45°C. The waxed specimens were transferred to the center of the glass slide, remaining water was removed, and the specimens were dried in an oven at 60°C for 30 min. The distal colon specimens were stained with Hematoxylin-eosin (HE) for immunohistochemistry and immunofluorescence studies. The proximal colon specimen was stained with toluidine blue for pathological observation of colon and mast cell counting.

#### 2.4.4 Mast Cell Counting and Colon Inflammation Observation

Mast cells were counted after toluidine blue staining. HE staining was used to observe colon inflammation in the rats.

### 2.5 Pharmacokinetic Study

After successful model establishment, rats in the model group were used to study the pharmacokinetics after single dose administration and multiple dose administration of WJW. In the single-dose administration, the rats were intragastrically administered 1 ml/100 g of the drug (629.6 mg/kg, which was 4 times the clinical dosage and an equivalence of 88 mg/kg berberine, 21.1 mg/kg palmatine, 0.22 mg/kg evodiamine, 0.28 mg/kg rutaecarpine, and 25.1 mg/kg paeoniflorin). For multiple-dose administration, the drug was given once a day for six consecutive days at the same concentration as the single-dose administration. At 12 h post-administration on day 6, jugular vein cannulation was performed on the rats. 12 h after the operation, the rats were intragastrically administered 1 ml/100 g WJW (629.6 mg/kg, an equivalence of 88 mg/kg berberine, 22.1 mg/kg palmatine, 0.22 mg/kg evodiamine, 0.28 mg/kg rutaecarpine, and 25.1 mg/kg paeoniflorin). Blood samples (200 μL) were taken from the jugular vein pre-administration and at 5 min, 15 min, 30 min, 1 h, 1.5, 2, 3, 4, 6, 8, 10, 12, 24, and 36 h post-administration. Samples were placed in heparin EP tubes, into which 200 μL heparin solution (50 IU/ml) was also added, and the tubes were centrifuged at 3,500 r/min for 15 min. Plasma (100 μL) was withdrawn and kept at −80°C. The amount of berberine, palmatine, evodiamine, rutaecarpine, and paeoniflorin in the rat plasma was determined simultaneously by the UPLC-MS/MS previously developed and validated (Zhang et al., 2014). Briefly, double internal standards including diphenhydramine hydrochloride and geniposide were selected and the positive and negative ion modes were used for simultaneous monitoring. Quantitation was performed using multiple-reaction monitoring (MRM) of the protonated molecular ion to predominant product ion pair, m/z 335 > 320 for berberine, m/z 352 > 336 for palmatine, m/z 304 > 134 for evodiamine, m/z 288 > 115 for rutaecarpine, m/z 256 > 152 for diphenhydramine hydrochloride detected in positive ion mode, and m/z 525 > 449 for paeoniflorin, m/z 387 > 225 for geniposide in negative ion mode. An aliquot of 100 μL plasma sample was spiked with 20 μL of the mixed internal standard working solution of 5 μg/ml diphenhydramine hydrochloride and 10 μg/ml geniposide and vortexed briefly. Then the mixture was added to 360 μL of acetonitrile to be deproteinized, mixed by vortex for 5 min and centrifuged at 13,000 rpm for 15 min at 4°C. The supernatant was evaporated by a gentle stream of nitrogen gas. The residue was reconstituted in 200 μL of the mobile phase followed by centrifugation at 13,000 rpm for 15 min. The supernatant was transferred into an autosampler vial and an aliquot of 3 μL was subsequently injected into the UPLC-MS/MS system for assay. The lower limit of quantification was established at 0.32 ng/ml for berberine, palmatine, evodiamine, and rutaecarpine, and 0.63 ng/ml for paeoniflorin in plasma.

The pharmacokinetic software WinNonlin 6.3 (Phoenix, Pharsight, United States) was used to plot the mean plasma concentration (ng/ml) vs. time profile for the above drug components. Non-compartmental analysis was performed to find the pharmacokinetic parameters under single-dose and multiple-dose administration, including terminal elimination half-life (T_1/2_), area under the plasma concentration vs. time curve from zero to last sampling time (AUC_0–t_), volume of distribution (V_d_/F), and total body clearance (CL/F). The peak plasma concentration (C_max_) and the time to reach C_max_ (T_max_) were read directly from the observed individual plasma concentration-time data. The pharmacokinetic parameters of the representative components of WJW were compared between the normal group and the model group under single high-dose and multiple high-dose scenarios.

After the blood collection at 36 h, the rats were sacrificed. Two colon sections were quickly cut out. One section was fixed in 2.5% glutaraldehyde solution, and the other in 4% paraformaldehyde. Both samples were embedded in paraffin and sliced. The ultrastructure of rat colon epithelial tight junctions was observed under a transmission electron microscope. The expression of tight junction proteins (Occludin and ZO-1), MLCK, and transport proteins (P-gp, MRP1, and MRP2) in the rat colon was detected by immunohistochemistry and immunofluorescence. The mechanism behind the PK difference of the five representative WJW components between the normal rats and CVH-IBS model rats was explored.

### 2.6 Immunohistochemistry Detection of *c-fos* Expression, Tight Junction Protein (ZO-1 and Occludin), MLCK, and Transporters (P-gp, MRP1, and MRP2) Expression in the Colon

For immunohistochemistry, colon tissue sections were deparaffinized with xylene and rehydrated in a gradient ethanol finishing in phosphate-buffered saline. Endogenous peroxidases were quenched using a few drops of H_2_O_2_. A citrate buffer solution was used to restore the antigens, and the colon tissues were then blocked with goat serum. After the sections were incubated with the primary antibody (*c-fos* rabbit anti-mouse polyclonal antibody, ZO-1 rabbit anti-mouse polyclonal antibody, Occludin rabbit anti-mouse polyclonal antibody, MLCK rabbit anti-mouse polyclonal antibody, P-gp rabbit anti-mouse polyclonal antibody, MRP1 rabbit anti-mouse polyclonal antibody, and MRP2 rabbit anti-mouse polyclonal antibody) overnight, the secondary antibody was applied. Then, the sections were washed with phosphate-buffered saline, diaminobenzidine was added for color rendering, and counterstaining was completed with hematoxylin. For immunohistochemical staining, the average integrated positive area from nine randomly chosen regions was calculated using Image Pro Plus 5.0 image analysis software. Brown coloration indicated positive expression.

### 2.7 Immunofluorescence Detection of Tight Junction Protein (ZO-1, Occludin) and MLCK Expression in the Colon

For immunofluorescence, following antigen repair in a 0.01 M citrate buffer, the tissues sections were blocked in goat serum. The tissue slices were incubated for 1 h with primary antibodies (ZO-1 rabbit anti-mouse polyclonal antibody, Occludin rabbit anti-mouse polyclonal antibody, and MLCK rabbit anti-mouse polyclonal antibody) followed by incubation for 1 h with secondary antibodies. The slices were stained with DAPI. Fluorescence was examined under an Olympus FV-1000 laser scanning confocal microscope. Red coloration indicated positive expression.

### 2.8 Statistical Analysis

All data were expressed as mean ± standard deviation. SPSS 17.0 statistical software was used for one-way analysis of variance and comparison between groups. The least significant difference test was used if homogeneity of variance was assumed for the samples, otherwise Dunnett’s T3 test was used. Significant difference was indicated as *p* < 0.05.

## 3 Results

### 3.1 Body Mass Change Over Time

**FIGURE 1:**
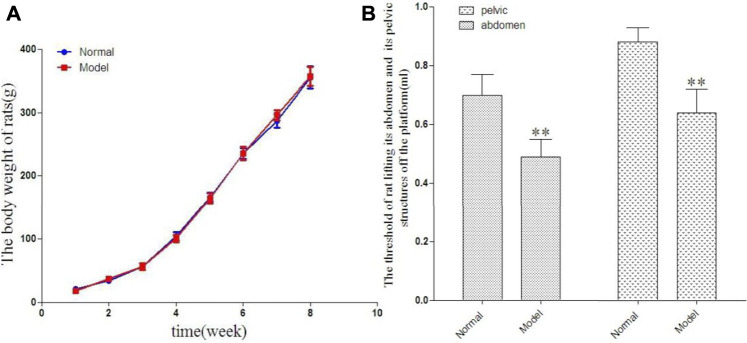
The change of rat in body weight in 8 weeks [**(A)**, g] and the threshold of rat lifting its abdomen off the platform and its pelvic structures off the platform [**(B)**, ml].

The rats in each group were weighed once a week during the course of their growth. There was no significant difference in body weight between the two groups at the corresponding time points. The results are shown in Figure 1A.

### 3.2 Comparison of Abdominal Lifting Threshold and Pelvic Lifting Threshold Between the Two Groups

Compared with the normal group, the abdominal lifting threshold and pelvic lifting threshold in the model group showed statistically significant decrease. The results are shown in Figure 1B.

### 3.3 Colon Morphology Change

**FIGURE 2:**
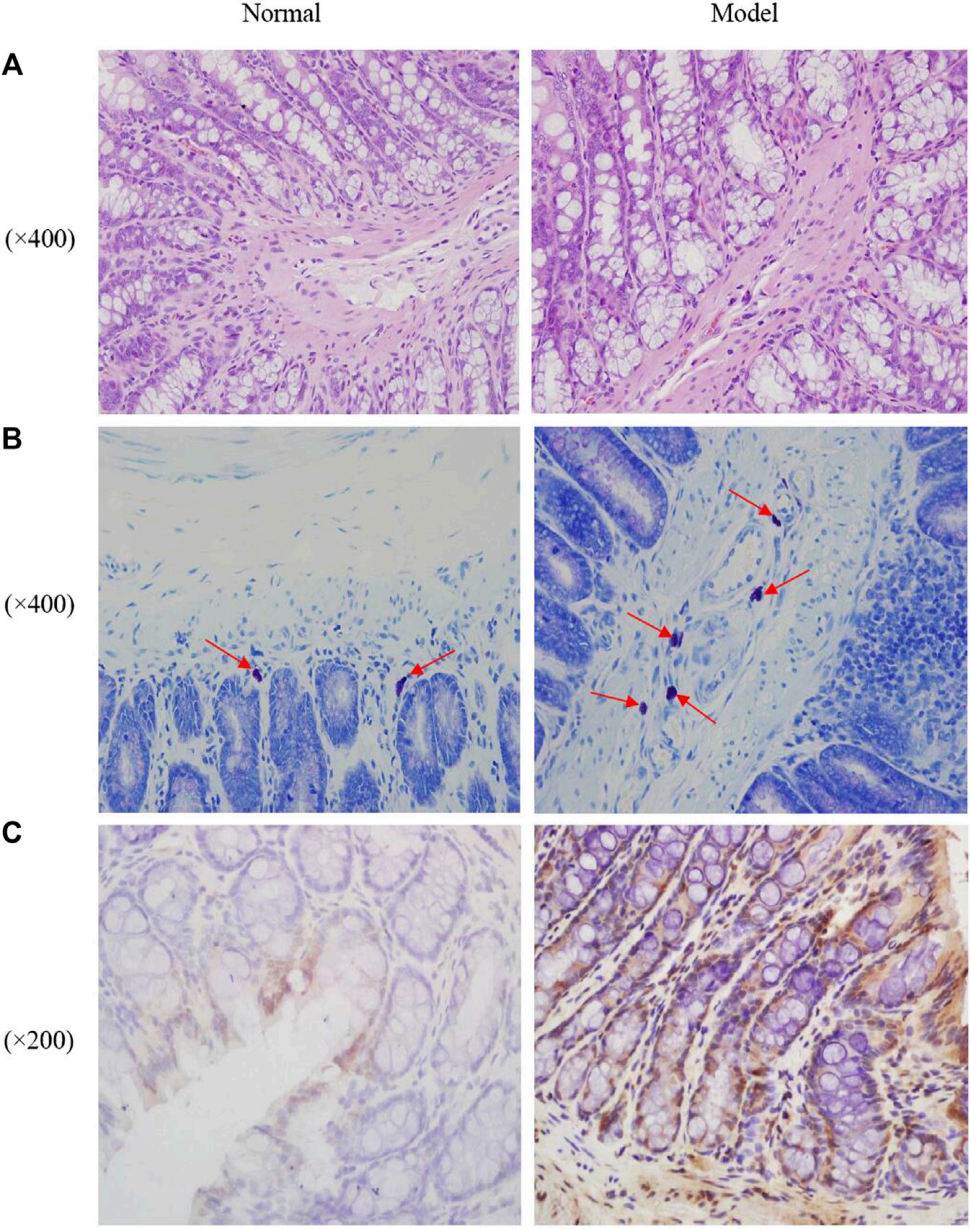
Photomicrographs of distal colons by HE staining **(A)**, mast cell in proximal colons by toluidine blue staining [**(B)**, the red arrows indicated the mast cells] and the expression of *c-fos* in the rats colon **(C)**.

As shown in Figure 2A, the colonic mucosa was complete with intact and continuous epithelium. The crypts were arranged in an orderly manner and their structure was discernable. No anomalies in cell morphology were observed. There was a small number of inflammatory cells infiltrating the lamina propria. There were no obvious inflammatory signs in the normal group or the model group.

### 3.4 Mast Cell Count in the Proximal Colon

As shown in Figure 2B; Table 1, mast cells were mainly distributed in the submucosa and muscularis mucosae. They appeared in groups or rows, or were scattered around blood vessels, lymphatic vessels, and peripheral nerves. The cells were round, oval, or irregular, with blue nuclei and purplish red particles randomly distributed in the cytoplasm around the nuclei. Compared with the normal group, a significantly higher number of mast cells was observed in the colonic submucosa of the model group.

**TABLE 1:** The number of the mast cells in proximal colon (mean ± SD, *n* = 5).

Group	Mast cell count (piece)
Normal	2.61 ± 0.86
Model	8.02 ± 1.63**

**p < 0.01 compared with normal group.

### 3.5 Immunohistochemistry Detection of *c-fos*

The expression of the proto-oncogene *c-fos* in rat colon tissue was quantified by immunohistochemistry. Expression of *c-fos* was significantly higher in the model group than in the normal group. The results are shown in Figure 2C; Table 2.

**TABLE 2:** The expression of *c-fos* in the rats colon.

Group	AIOD
Normal	843.6 ± 137.5
Model	3,267.0 ± 627.9**

**p < 0.01 compared with normal group.

### 3.6 Pharmacokinetic Results

**FIGURE 3:**
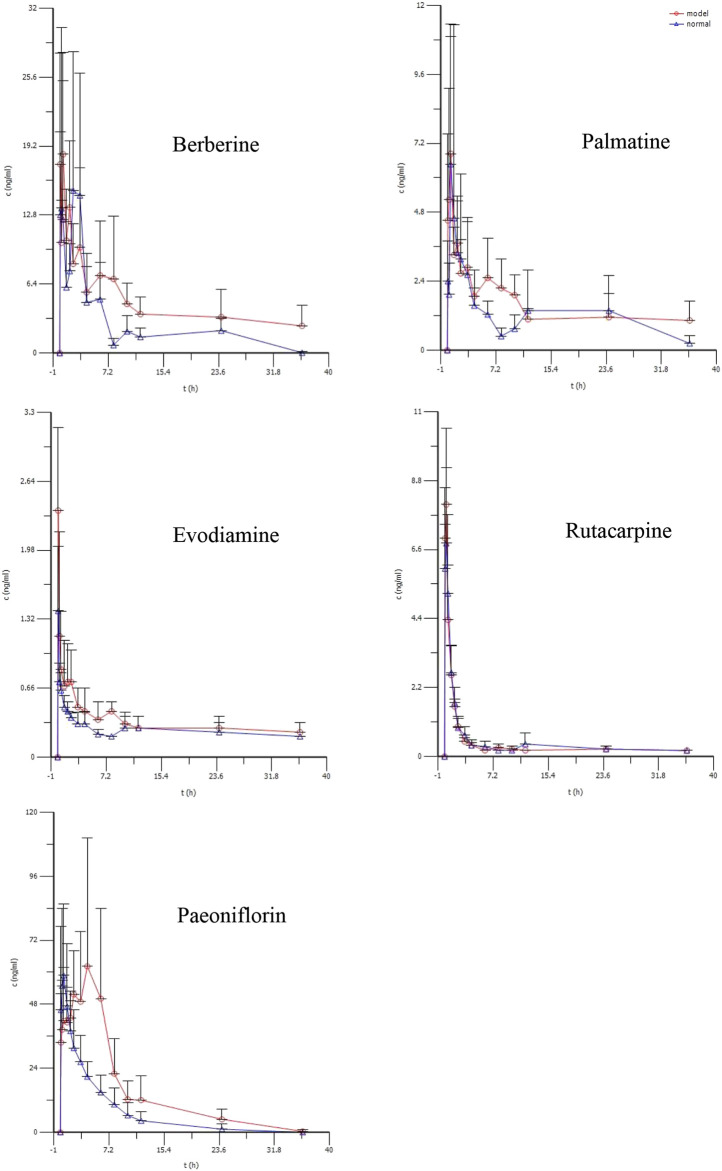
The mean plasma concentration (ng/ml) of berberine, palmatine, Evodiamine, rutacarpine, and paeoniflorin vs. time(h) profiles after single doses of oral administration of WJW Extract (629.6 mg/kg) with the equivalent dose of 88.4 mg/kg, 21.1 mg/kg, 0.22 mg/kg, 0.28 mg/kg, 25.1 mg/kg for berberine, palmatine, evodiamine, rutacarpine, and paeoniflorin respectively in normal and CVH-IBS model rats. Values were expressed as mean ± SD (*n* = 5).

The average plasma concentration profile of the five representative components (berberine, palmatine, evodiamine, rutaecarpine, and paeoniflorin) of WJW after single dose intragastric administration is shown in Figure 3, and the pharmacokinetic parameters are presented in Table 3. For berberine and palmatine, T_1/2_ and AUC_0–t_ were significantly higher in model rats than in normal rats, while Cl/F was significantly lower. No significant differences were observed in T_max_, C_max_, and V_d_/F. For evodiamine, C_max_ and AUC_0–t_ were significantly higher in model rats than in normal rats, while no significant differences were observed in T_1/2_, T_max_, V_d_/F, and Cl/F. For rutaecarpine, there was no significant difference in the pharmacokinetic parameters between model rats and normal rats. For paeoniflorin, T_max_ and AUC_0–t_ were significantly higher in model rats than in normal rats, while Cl/F was significantly lower. No significant difference was observed in T_1/2_, C_max_, and V_d_/F.

**TABLE 3:** Pharmacokinetic parameters of berberine, palmatine, Evodiamine, rutacarpine, and paeoniflorin in rat after single doses of oral administration of WJW Extract (629.6 mg/kg) with the equivalent dose of 88.4 mg/kg, 21.1 mg/kg, 0.22 mg/kg, 0.28 mg/kg, 25.1 mg/kg for berberine, palmatine, evodiamine, rutacarpine, and paeoniflorin, respectively (mean ± SD, *n* = 5).

Parameters	Berberine		Palmatine		Evodiamine		Rutacarpine		Paeoniflorin	
	Normal	Model	Normal	Model	Normal	Model	Normal	Model	Normal	Model
T_1/2,λz_ (min)	562.5 ± 237.2	1,384.9 ± 712.4*	733.8 ± 67.4	1,532.4 ± 612.7*	1,808.4 ± 621.2	1,477.3 ± 341.0	977.5 ± 394.4	774.0 ± 293.5	248.1 ± 101.9	335.2 ± 45.4
T_max_ (min)	36.3 ± 16	30.0 ± 0.0	37.5 ± 15.0	26.3 ± 7.5	5.0 ± 0.0	5.0 ± 0.0	15.0 ± 0.0	15.0 ± 0.0	21.0 ± 8.2	80.0 ± 45.8*
C_max_ (ng/ml)	27.5 ± 10.2	21.8 ± 8.4	8.3 ± 4.3	8.9 ± 3.0	1.4 ± 0.6	2.4 ± 0.7*	7.5 ± 5.1	8.9 ± 6.8	69.4 ± 24.0	85.9 ± 36.7
AUC_0–t_ (min.ng/ml)	5,443.0 ± 1,405.8	9,930.8 ± 2,304.5**	2,365.5 ± 410.6	3,527.0 ± 717.8**	573 ± 45.3	733.9 ± 160.2*	989.9 ± 293.0	894.9 ± 251.4	15,428.9 ± 5,063.6	33,140.6 ± 5,613.9**
V_d_/F_,λz_ (L/kg)	9,597.4 ± 1960.5	10,972.4 ± 3,312.9	7,523.3 ± 1,692.1	6,760.2 ± 919.9	513.6 ± 76.7	431.9 ± 145.4	351.5 ± 57.9	332.5 ± 156.5	543.4 ± 213.9	387.5 ± 93.0
Cl/F (L/h/kg)	840.7 ± 250.8	397.3 ± 142.7**	427.7 ± 89.4	288.9 ± 114.4*	12.4 ± 1.9	10.2 ± 3.2	12.7 ± 4.0	13.4 ± 4.9	110.5 ± 48.1	43.3± 9.5**

*p < 0.05 compared with normal group, **p < 0.01 compared with normal group.

**FIGURE 4:**
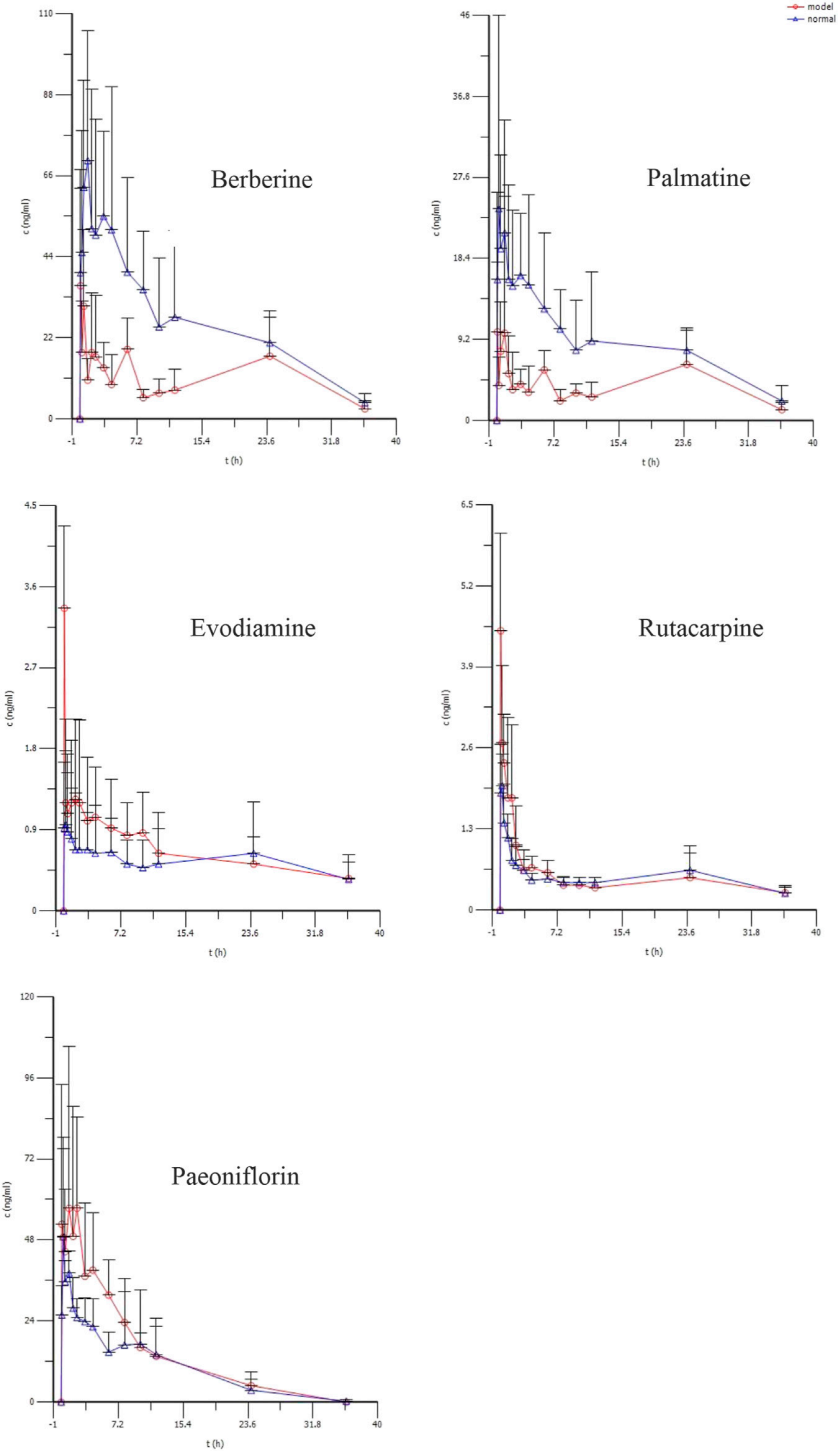
The mean plasma concentration (ng/ml) of berberine, palmatine, Evodiamine, rutacarpine, and paeoniflorin vs. time (h) profiles after multiple doses of oral administration of WJW Extract (629.6 mg/kg) with the equivalent dose of 88.4 mg/kg, 21.1 mg/kg, 0.22 mg/kg, 0.28 mg/kg, 25.1 mg/kg for berberine, palmatine, evodiamine, rutacarpine, and paeoniflorin respectively in normal and CVH-IBS model rats. Values were expressed as mean ± SD (*n* = 5).

The average plasma concentration profile of the five representative components of WJW after multiple dose intragastric administration is shown in Figure 4, and the pharmacokinetic parameters are given in Table 4. The results showed that for berberine, T_1/2_, V_d_/F, and Cl/F were significantly higher in model rats than in normal rats, while C_max_ and AUC_0–t_ were significantly lower. No significant difference was observed in T_max_. For palmatine, T_1/2_, V_d_/F, and Cl/F were significantly higher in model rats than in normal rats, while C_max_ and AUC_0–t_ were significantly lower. No significant difference was observed in T_max_. For evodiamine, C_max_ and AUC_0–t_ were significantly higher in model rats than in normal rats, while T_1/2_, T_max_, and V_d_/F were significantly lower. No significant difference was observed in Cl/F. For rutaecarpine, C_max_ was significantly higher in model rats than in normal rats, while T_max_ was significantly lower. No significant differences were observed in T_1/2_, AUC_0–t_, V_d_/F, and Cl/F. For paeoniflorin, T_max_ was significantly higher in model rats than in normal rats, while no significant difference was observed in T_1/2_, C_max_, AUC_0–t_, V_d_/F, and Cl/F.

**TABLE 4:** Pharmacokinetic parameters of berberine, palmatine, Evodiamine, rutacarpine, and paeoniflorin in rat after multiple doses of oral administration of WJW Extract (629.6 mg/kg) with the equivalent dose of 88.4 mg/kg, 21.1 mg/kg, 0.22 mg/kg, 0.28 mg/kg, 25.1 mg/kg for berberine, palmatine, evodiamine, rutacarpine, and paeoniflorin, respectively (mean ± SD, *n* = 5).

Parameters	Berberine		Palmatine		Evodiamine		Rutacarpine		Paeoniflorin	
	Normal	Model	Normal	Model	Normal	Model	Normal	Model	Normal	Model
T_1/2,λz_ (min)	559.5 ± 118.8	970.4 ± 211.3**	706.78 ± 249.4	1,376.6 ± 102.4**	1,967.4 ± 499.3	919.3 ± 237.2**	1,370.8 ± 276.1	1,287.2 ± 465.4	386.2 ± 141.2	468.9 ± 156.3
T_max_ (min)	52.5 ± 15.0	5.0 ± 0.0**	20 ± 8.7	13.3 ± 14.4	16.7 ± 11.3	5.0 ± 0.0*	10.0 ± 5.5	5.0 ± 0.0*	18 ± 6.7	105.0 ± 30.0**
C_max_ (ng/ml)	85.6 ± 20.6	48.2 ± 18.2	34.9 ± 14.5	16.8 ± 10.9*	1.1 ± 0.7	3.5 ± 2.5*	2.2 ± 1.2	5.5 ± 2.9*	54.7 ± 16.1	77.8 ± 38.1
AUC_0–t_ (min.ng/ml)	58,252.1 ± 9,131.6	26,206.2 ± 7,505.8**	19,125.1 ± 3,519.3	10,122.9 ± 1,371.9**	857.1 ± 303.9	1,541.3 ± 602.8*	1,153.0 ± 338.5	1,169 ± 348.8	21,785.4 ± 1719.3	26,442.9 ± 6,743.5
V_d_/F_,λz_ (L/kg)	1,185.1 ± 360.9	4,189.1 ± 613.2**	1,154.4 ± 335.1	5,545.8 ± 4,148.8*	383.7 ± 147.6	178.3 ± 61.4**	375.7 ± 109.1	323.7 ± 122.5	550.8 ± 128.7	485.9 ± 67.9
Cl/F (L/h/kg)	87.1 ± 13.3	156.9 ± 48.2**	60.2 ± 10.6	162.1 ± 108.9*	11.5 ± 5.8	7.4 ± 2.0	9.6 ± 2.9	10.4 ± 2.7	63.9 ± 11.8	50.6 ± 16.9

*p < 0.05 compared with normal group, **p < 0.01 compared with normal group.

### 3.7 Electron Microscopy Observation

**FIGURE 5:**
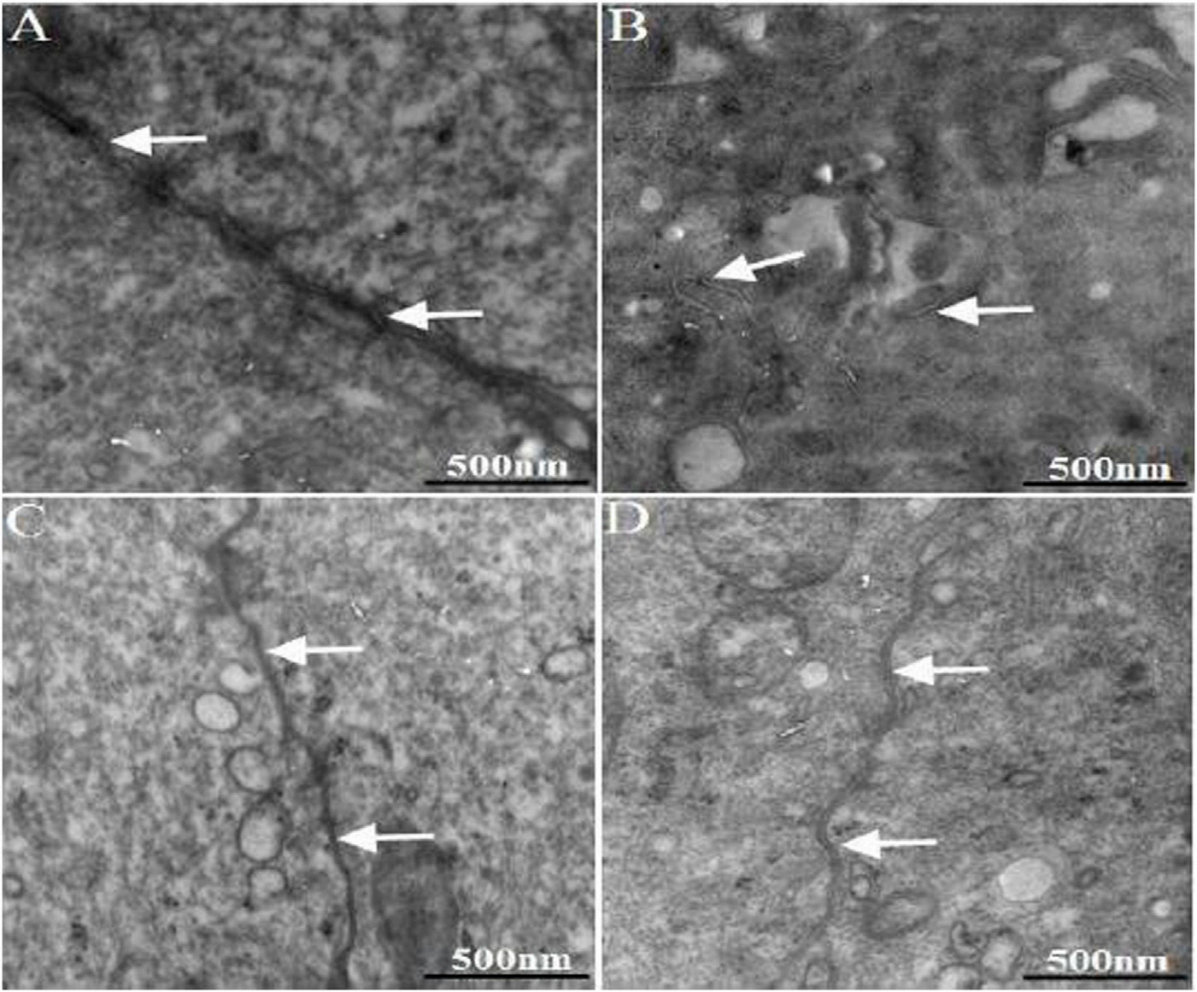
The ultra-structure of TJ in the colonic epithelium of rats. The arrows indicated TJ [**(A)**: normal; **(B)** model; **(C)** multiple dose of WJW in normal; **(D)** multiple dose of WJW in model].

The ultrastructure of rat colon tissue epithelial tight junctions was observed under transmission electron microscope. The results are shown in Figure 5. The epithelial tight junctions of normal rat colon showed a complete structure, featuring compact and well-sealed junctions. In the model group, the colon epithelial tight junctions appeared damaged. The junctions were loose, and showed wide gaps and reduced junction density. 1 week after WJW administration, the tight junctions of the model group regained their integrity compared with pre-administration time, with re-established junctions.

### 3.8 Immunohistochemistry Detection of *c-fos*, Tight Junction Protein (ZO-1 and Occludin), MLCK, and Transporters (P-gp, MRP1, and MRP2) Expression in the Colon

**FIGURE 6:**
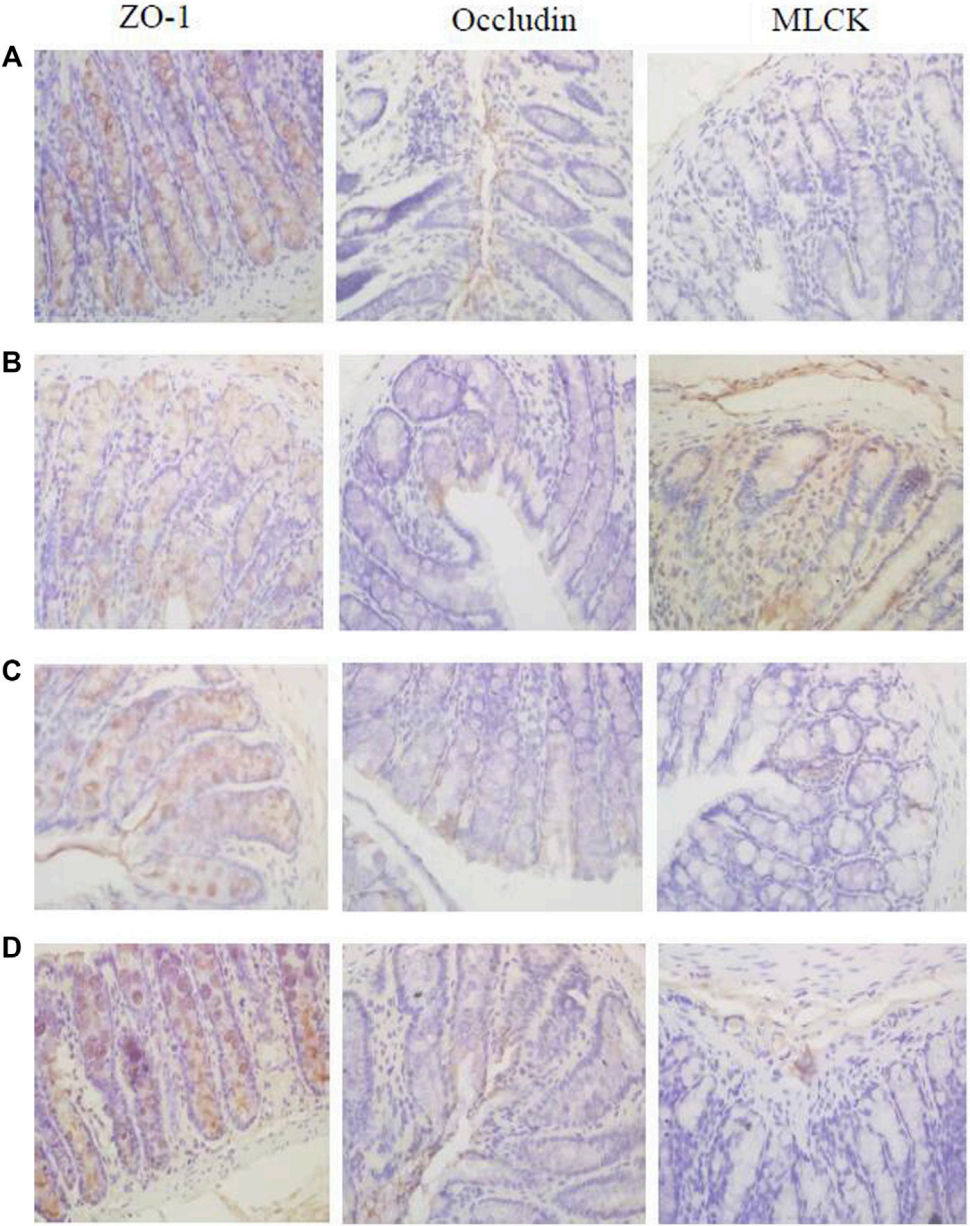
The expression of ZO-1, Occludin, and MLCK in the rats colon [**(A)**: normal; **(B)** model; **(C)** multiple dose of WJW in normal; **(D)** multiple dose of WJW in model].

**FIGURE 7:**
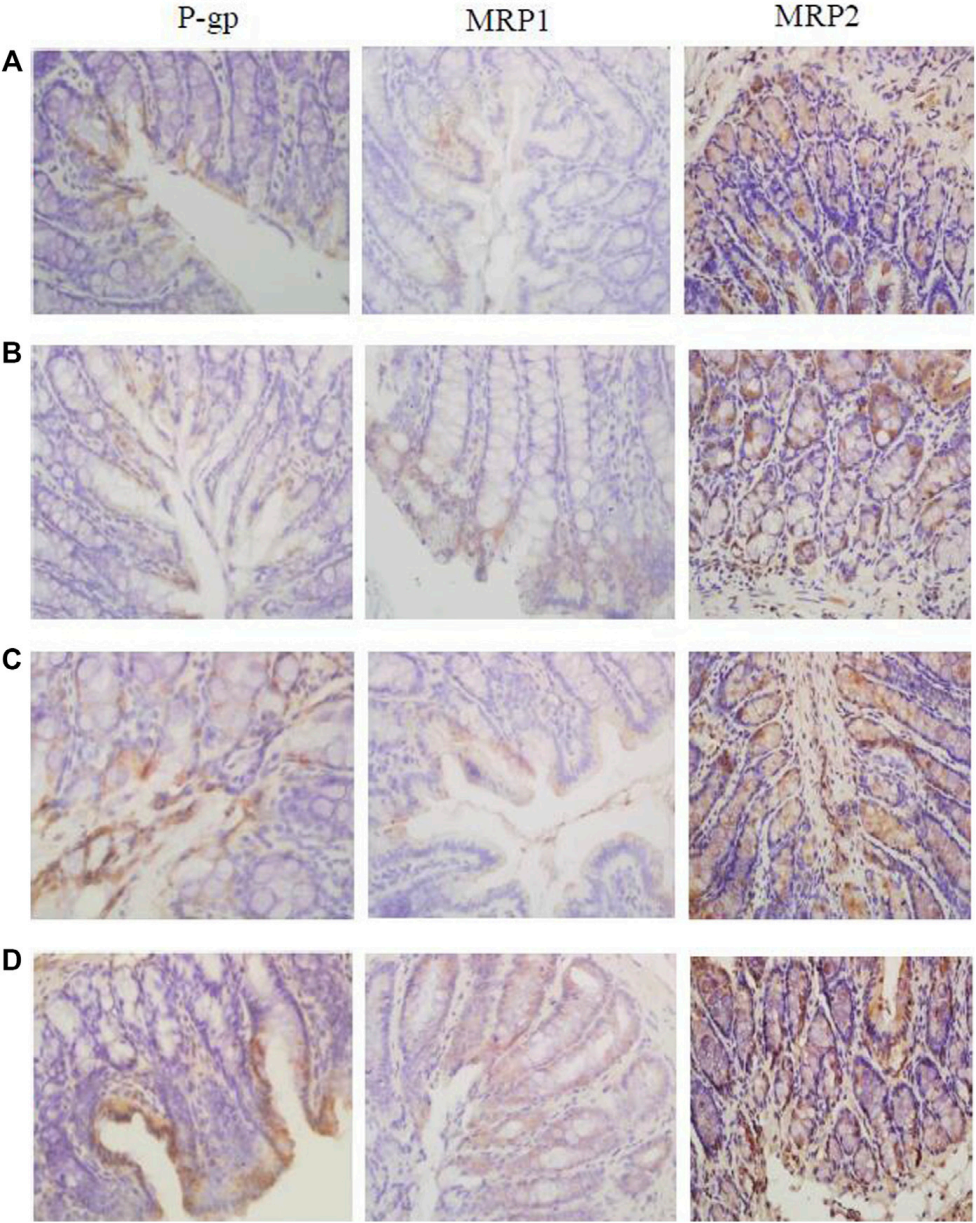
The expression of P-gp, MRP1, and MRP2 in the rats colon [**(A)**: normal; **(B)** model; **(C)** multiple dose of WJW in normal; **(D)** multiple dose of WJW in model].

Immunohistochemistry results showed that the expression levels of tight junction proteins (Occludin and ZO-1) were significantly lower in the colons of CVH-IBS model rats compared with normal rats. After multiple-dose administration, the expression in model rats increased significantly compared with pre-administration, but was still lower than that in the normal group. No significant change post-administration was found in normal rats. MLCK expression was significantly higher in the colons of model rats compared with normal rats. After multiple-dose administration, the expression in model rats decreased significantly compared with pre-administration, but was higher than that in the normal group. No significant change post-administration was found in normal rats. The expression of transporters (P-gp, MRP1, and MRP2) did not significantly differ in the colon tissue of model rats compared with normal rats. After multiple-dose administration, their expression in both model rats and normal rats was significantly higher than that before administration. The expression following multiple-dose administration was significantly higher in the model group than in the normal group. The results are shown in Figures 6, 7; Table 5.

**TABLE 5:** The expression of ZO-1, Occludin, MLCK, P-gp, MRP1, and MRP2 in the rats colon (*n* = 5, $\bar{x}$ ± s, AIOD).

Group	Normal	Model	Normal + WJW	Model + WJW
ZO-1	6,714.0 ± 396.5	3,371.0 ± 650.7**	6,983.0 ± 702.6	5,614.2 ± 382.5**^##△△^
Occludin	3,860.6 ± 678.0	1,615.2 ± 447.3**	3,954.4 ± 258.5	2,709.6 ± 252.5**^##△△^
MLCK	959.8 ± 362.7	4,131.8 ± 500.2**	1,002.6 ± 166.0	2,091.0 ± 492.5**^##△△^
P-gp	2,927.6 ± 258.2	2,843.8 ± 314.1	5,823.0 ± 800.4**	6,695.0 ± 410.8**^##△^
MRP1	2,878.4 ± 462.1	2,702.8 ± 429.2	3,905.8 ± 356.4**	4,754.4 ± 467.7**^##△△^
MRP2	4,551.8 ± 627.1	4,424.6 ± 660.1	6,680.4 ± 509.9**	7,459.4 ± 446.1**^##△^

**p < 0.01 compared with normal group, ^*##*^ *p* < 0.01 compared with model group, ^△^ *p* < 0.05, and ^△△^ *p* < 0.01 compared with normal + WJW group.

### 3.9 Immunofluorescence Detection of Tight Junction Protein (ZO-1 and Occludin) and MLCK-1 Expression in Rat Colon

**FIGURE 8:**
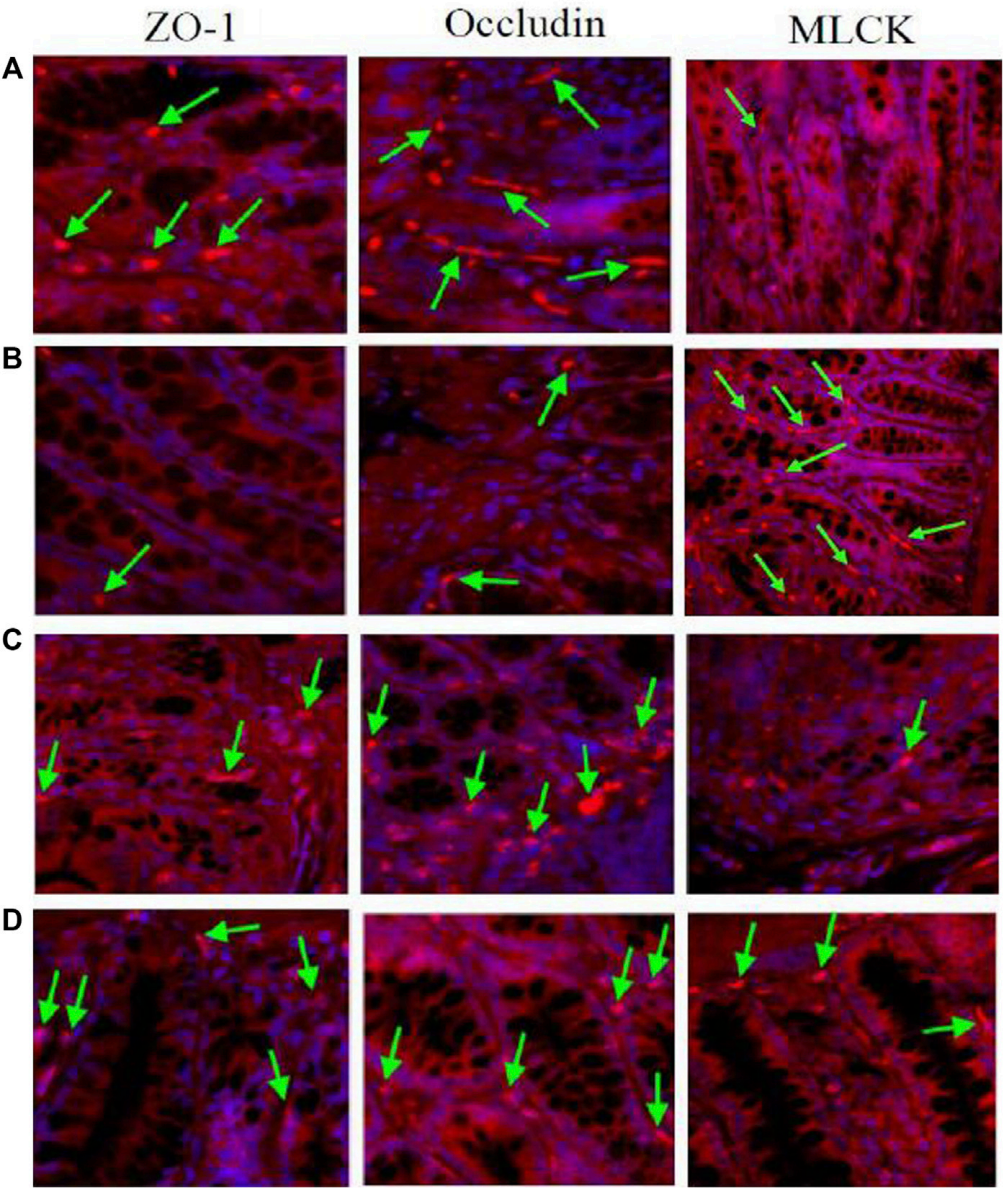
The expression of TJ proteins (Occludin and ZO-1) and MLCK in the rats colon. The arrows indicated the TJ proteins or MLCK [**(A)**: normal; **(B)** model; **(C)** multiple dose of WJW in normal; **(D)** multiple dose of WJW in model].

Immunofluorescence results showed that the expression of tight junction proteins (Occludin and ZO-1) was lower in the colon of model rats compared with normal rats, while the expression of MLCK was higher. After multiple-dose administration, the expression of tight junction proteins in model rats increased significantly compared with pre-administration, while MLCK expression decreased significantly. No significant differences were found in tight junction protein and MLCK expression in normal rats before and after multiple-dose administration. After multiple-dose administration, the expression of tight junction proteins in the model group was lower than that in the normal group, while the expression of MLCK expression was higher. The results are shown in Figure 8.

## 4 Discussion

IBS is a common digestive system condition. Its causes and pathogenesis are not yet well-understood, and effective therapeutic drugs are lacking. In recent years, TCM and its compound prescriptions have been playing an increasingly important role in the treatment of CVH-IBS. An appropriate animal model is indispensable in the screening and evaluation of TCM and its compound prescriptions in the treatment of CVH-IBS. The CVH-IBS rat model established in this study closely simulated the main symptoms of CVH-IBS patients, including the significantly reduced abdominal lifting threshold and pelvic lifting threshold, the lack of apparent colon inflammation, significantly elevated *c-fos* expression, and hypersensitivity towards pain. For these reasons, the established model is applicable as a CVH-IBS rat model for experimental studies.

Following the successful establishment of the CVH-IBS rat model, the pharmacokinetics of WJW in normal rats and model rats after single-dose administration was compared. The results showed that compared with the normal group, the absorption of berberine, palmatine, and paeoniflorin increased in model rats after intragastric administration of the drug, while the elimination of these components slowed. The absorption of evodiamine increased in model rats compared with normal rats, with no significant difference observed in elimination. There was no significant difference in the absorption and elimination of rutaecarpine in model rats compared with normal rats.

The pharmacokinetics of drugs, besides being influenced by physiological state and pathological states, may also be affected by number of doses. Therefore, our study also compared the pharmacokinetics of WJW in normal rats and CVH-IBS rats after 7 days of continued administration. The results showed that the absorption of berberine and palmatine was reduced in model rats compared with normal rats, while the elimination was accelerated. The absorption of evodiamine in model rats increased compared with that in normal rats, with no significant change observed in elimination. No significant difference was found in the absorption and elimination of rutaecarpine and paeoniflorin. These results are in stark contrast to those from single-dose administration.

Hence, pharmacokinetic differences for the representative components of WJW were confirmed between normal rats and CVH-IBS model rats following both single-dose administration and multiple-dose administration. The reason for the differences, however, is still unknown. It is well known that the pharmacokinetic process of drug include absorption, distribution, metabolism, and excretion (ADME). The tight junctions (TJ) between cells and transporters could affect the absorption of drugs. Transporters influence the disposition of drugs within the body by participating in ADME (Klaassen and Aleksunes, 2010), and is one of important mechanisms for herb–drug interactions (Choi, et al., 2011). Transporters mainly include influx proteins (organic anion transporters and organic cation transporters, etc.,) and efflux transporters (P-gp, MRP1, and MRP2, etc.). Moreover, the literature reports that berberine is a substrate of P-gp and MRP2 (Pan et al., 2002; Maeng et al., 2002; Qian et al., 2016; Wang et al., 2007). Also, palmatine and paeoniflorin are also substrates of P-gp (Liu et al., 2006; Zhang et al., 2014). In addition, the study of TJ was carried out from the perspective of increasing absorption, so only common efflux transporters (P-gp, MRP1, and MRP2) were selected for transporters, and influx transporters were not involved.

Previous studies have shown that a healthy intestinal mucosal barrier mainly consists of a mechanical, chemical, immunological, and biological barriers, of which the mechanical barrier is the most important (Assimakopoulos,et al., 2011). Under normal physiological conditions, the junction complexes between adjacent cells of intestinal epithelium form a highly selective mechanical barrier, allowing passage of only small-molecular, water-soluble substances such as water and ions *via* passive diffusion, while preventing the entrance of macromolecular antigens through the epithelium to reach the submucosa and incite an immune response (Fernandez-Blanco et al., 2011). A junction complex is made of TJ, gap junctions, adhesion junctions, and desmosomes. TJ are located at the apex of the junction complex and serves as the most important connections between cells. They are a type of dynamic structure consisting of various tight junction proteins, including transmembrane proteins, such as occludin and claudins, junction adhesion molecules, and proteins such as ZOs and 7H6 involved in the assembly of the tight junction at the cytoplasmic surface. Of these, occludin and ZO-1 are key to maintaining the normal structure and function of tight junctions (Wu,et al., 2000).

MLCK is a Ca^2+^/calmodulin-dependent enzyme located in the cell membrane. It regulates the phosphorylation of myosin light chain, as well as the cytoskeleton (Turner,et al., 1997; Kevin and Jerold, 2012). MLCK has an important function in maintaining the integrity of tight junctions (Shen and Turner, 2006). Because of their compact nature, the destruction of tight junctions inevitably affects the permeability of intestinal mucosa. It has been found that intestinal mucosal permeability is increased (Barbara, 2006; Barbara,et al., 2009), while the expression of Occludin and ZO-1 is decreased in IBS patients compared with healthy individuals (Zeng,et al., 2008). Some studies have suggested the downregulation and redistribution of tight junction proteins as the molecular mechanism behind the intestinal mucosal permeability changes, which affect the absorption of drugs.

In our study, immunohistochemistry results showed that compared with the normal group, the expression of tight junction proteins (Occludin and ZO-1) decreased in the model group, the expression of MLCK increased, and there was no significant difference in the expression of transporters (P-gp, MRP1, and MRP2). Immunofluorescence results also showed that compared with the normal group, the expression of tight junction proteins decreased in the model group, and the expression of MLCK increases, leading to the increased permeability of colon mucosa in the model group and increased drug absorption. This explains to some extent the increased absorption of berberine, palmatine, evodiamine, and paeoniflorin in CVH-IBS rats after the single-dose administration of WJW.

Following the multiple-dose administration of WJW, the expression of tight junction proteins and transport proteins increased significantly in the model group compared with pre-administration, while the expression of MLCK decreased significantly. For the normal group after multiple-dose administration, no differences were observed in tight junction protein and MLCK expression levels, while the expression of transport proteins significantly increased. This shows the ability of WJW to promote tight junction protein expression and protect the intestinal mucosa. The drug also increased the expression of transporters probably *via* the induction by berberine, palmatine, and paeoniflorin, as they are the substrates of these transporters.

However, comparing the model group and the normal group after multiple-dose administration of WJW, the expression of tight junction proteins (Occludin and ZO-1) was lower in the model, the expression of MLCK was higher, and the expression of transporters (P-gp, MRP1, and MRP2) was significantly higher. These results suggest the removal of berberine and palmatine by the significantly higher level of transporters as a possible reason for the reduced absorption of these two drugs in CVH-IBS model rats compared with normal rats, after the multiple-dose administration of WJW. This effect dominates over the increased absorption by colon mucosa as a result of the permeability change.

It is well known that DMEs and transporters play important roles in the ADME processes. The roles of DMEs in pharmacokinetics have been extensively investigated for years (Zanger and Schwab, 2013). The metabolism of drugs in the body can be mediated by enzymes responsible for phase I (oxidation, reduction, and hydrolysis) and/or phase II (conjugation) biotransformation. Among these, CYP enzymes are well known for their roles in phase I oxidative metabolism (Jabir, et al., 2012; Kumar,et al., 2012; Zhou,et al., 2012); enzymes known for phase II metabolism include N-acetyl transferase, glutathione S-transferase, uridine 50- diphospho-glucuronosyltransferases, and sulfotransferase. Under certain pathological circumstances, the expression and activities of enzymes and transporters can be regulated, thereby leading to an alteration of the pharmacokinetic properties of substrate drugs (Wu and Lin., 2019). In the present study, it is worth mentioning that rutaecarpine differs from other four components after one intake of WJW. However, these results of tight junction and transporters could not explain the phenomenon. The reason for that rutaecarpine differs from other components is unclear. It has been reported P450 1A and 2B might predominantly metabolize rutaecarpine in rat liver microsomes (Lee, et al., 2004). It is still unclear if their quantity and activity change under the pathological state of IBS. Moreover, intestinal microbiome (Gill, et al., 2006; Men et al., 2015) also play a vital part in drug metabolism. Therefore, we propose as the next step in our research to examine the specific mechanisms behind the pharmacokinetic differences of the representative components of WJW between normal rats and IBS rats from the perspectives of intestinal microbiome and CYP enzymes.

## Data Availability

The original contributions presented in the study are included in the article/Supplementary Material, further inquiries can be directed to the corresponding authors.

## Abbreviations

ADME, absorption, distribution, metabolism and excretion; AUC_0–t_, area under the plasma concentration vs. time curve from zero to last sampling time; CL/F, total body clearance; C_max_, the peak plasma concentration; CVH-IBS, chronic visceral hypersensitivity irritable bowel syndrome; CYP, cytochrome P450; DMEs, drug-metabolizing enzymes; HE, hematoxylin-eosin; IBS, irritable bowel syndrome; MLCK, myosin light chain kinase; MRP1, multidrug resistance associated protein 1; MRP2, multidrug resistance associated protein 2; P-gp, P-glycoprotein; PTCA, percutaneous transluminal coronary angioplasty; T_1/2_, terminal elimination half-life; TCM, Traditional Chinese medicine; TJ, tight junctions; T_max_, the time to reach C_max_; V_d_/F, volume of distribution; WJW, Wuji Wan.
